# Threat of COVID-19 and emotional state during quarantine: Positive and negative affect as mediators in a cross-sectional study of the Spanish population

**DOI:** 10.1371/journal.pone.0235305

**Published:** 2020-06-25

**Authors:** María del Carmen Pérez-Fuentes, María del Mar Molero Jurado, África Martos Martínez, Jose Jesús Gázquez Linares

**Affiliations:** 1 Department of Psychology, Faculty of Psychology, University of Almería, Almería, Spain; 2 Department of Psychology, Faculty of Psychology, Universidad Politécnica y Artística del Paraguay, Asunción, Paraguay; 3 Department of Psychology, Universidad Autónoma de Chile, Providencia, Chile; University of Extremadura, SPAIN

## Abstract

**Aims:**

The objective of this study was therefore to analyze the effect of exceptionally stressful situations, such as the current health risk, on the cognitive and emotive state of the individual, that is, perceived threat and emotional state on affect and mood.

**Method:**

This was a cross-sectional study with snowball sampling. The sample came to 1014 Spanish adults (67.2% women and 32.8% men). The Perception of Threat from COVID-19 questionnaire, the Affective Balance Scale and the Mood Evaluation Scale were used.

**Results:**

The results showed that the perception of threat from COVID-19 was related positively to negative affect and emotional signs, that is, sadness-depression, anxiety and anger-hostility. There was a direct positive effect of perceived threat from COVID-19 on sadness-depression, anxiety and anger-hostility moods, while anxiety and anger-hostility had a direct positive effect on perception of threat from the virus. Thus, there was a circular relationship, in which perceived threat influenced the presence of negative mood, and negative mood, in turn, linked to emotions of irritation and agitation from a present situation, promoted the feeling of threat.

**Conclusions:**

A negative affective balance increases both one’s perception of threat from COVID-19 and negative mood. Thus, knowing the emotional and cognitive effects on the population would enable measures to be put into service to facilitate their effective coping.

## Introduction

Generalized transmission of the novel coronavirus called SARS-CoV-2 or COVID-19 detected in China in December 2019 has placed international public healthcare in check [1,2]. Within the Spanish borders, this has generated a scenario in which the response capacity of the healthcare system depends on the service of healthcare professionals and the disposition of the population to maintain the requirements of isolation, hygiene and social distancing to reduce exposure to contagion [3].

### Perception of threat from the disease

Beyond the danger to health, the presence of this state of emergency and constant worry increase stress factors, with a consequent increase in anxiety, and even development of emotional disorders [4,5]. The perception of threat to one’s health is based on perceived susceptibility (that is, belief of vulnerability to and possibility of contracting the disease) and in perceived severity (that is, beliefs related to changes that having the disease would cause in all areas of life) [6]. A state of extended hypervigilance, feelings of life-threatening danger and strong sensitivity to the appearance or recurrence of the disease are characteristic of those who show high perception of threat [7]. Thus, beyond physical health, people at risk and patients diagnosed with COVID-19 may experience fear from the consequences of infection, such as death or severe physical disability [8]. Such emotional disturbance combines with boredom, loneliness and anger that could appear in quarantine [9].

### Impact of quarantine on emotional state

Concern for one’s health, especially among those who live where the outbreak is worst, is combined with the home confinement decreed by the state of emergency [10]. Although there are significant gaps in treatment and primary prophylactic measures, such as vaccines, in this epidemic [11], the need for those at risk to be isolated is conclusive and requires a high level of coordination and citizen responsibility [12]. This confinement can generate negative consequences to physical and psychological wellbeing, such as anxiety and insomnia, promoted by alteration of physiological and circadian rhythms. However, of much greater concern is the psychological impact [13]. Stressful factors such as prolonged confinement, fear of infection, frustration, boredom, inadequate information, lack of contact with other persons outside of those with whom one lives, lack of personal space in the home and financial loss increase worry and individual perception of threat [14], especially when there is no way to cope constructively with the adversity [15]. In this situation of great stress and uncertainty, it is normal to develop a stronger perception of threat, characterized by assignment of negative meaning to originally neutral stimuli [16]. The perception of threat generates emotional and mood alterations and vice versa, such that the state of anxiety and changes in humor can also lead to heightened perception of threat [17].

In this case, sudden changes and unexpected situations, such as the population is now confronting with COVID-19, are perceived as physical or mental threats, challenging one’s ability to control the situation. Any situation creating an emotional impact dominating one’s usual coping strategies can generate anxiety and apprehension in individuals who normally would be mentally balanced and healthy [18]. With regard to emotional control and stress management, individual variables such as emotional intelligence, self-efficacy and optimism have been shown to be determinant [19,20,21,22]. Emotion regulation processes based on conscious emotional control facilitate positive affective experiences, contrary to automatic and preconscious, which are related to negative affect [23].

Referring specifically to the impact of confinement because of COVID-19, Moreira de Medeiros et al., [8] suggest that quarantine can generate feelings of boredom, loneliness and anger. Along this line, studies done in Asian populations, where isolation measures began much earlier than in Spain, show how individuals during the epidemic experienced a moderate or severe psychological impact and usually showed depressive symptoms and higher anxiety and stress [13].

**Fig 1:**
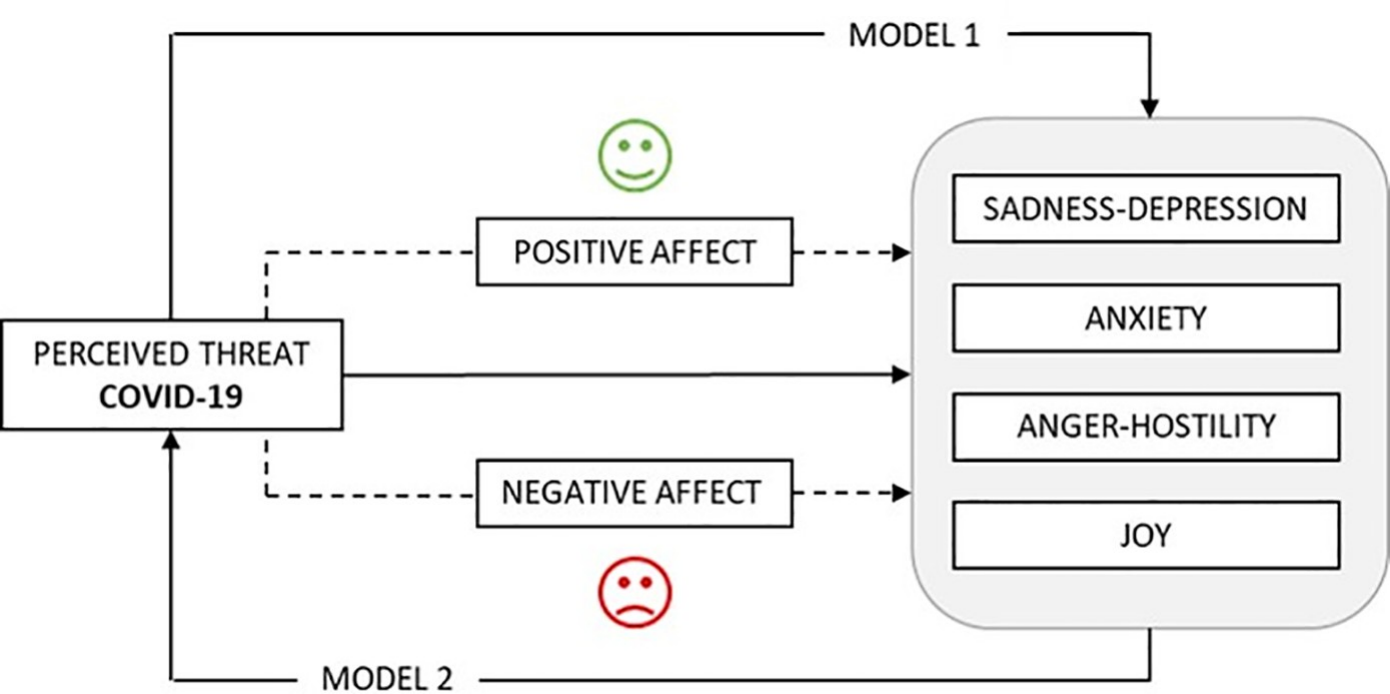
Hypothesized mediation models.

In view of the above, the objective of this study was to analyze the effect of exceptionally stressful situations, such as the current health risk, on the cognitive and emotional state of the individual, that is on the perception of threat and emotional state, both on effects and mood. The starting hypothesis was that perception of threat in the exceptional state of health emergency caused by COVID-19, affects one’s emotional situation (Model 1), and this, in turn, affects perception of risk (Model 2), in which positive and negative affect balances act as mediators in these relationships (Fig 1).

## Method

### Participants

The original sample of general Spanish population was *N* = 1043. Based on answers to control questions (CQ), those cases in which random or incongruent answers were detected were discarded (-29), leaving a final sample of 1014, all of them residents of Spain in 19 autonomous regions, but 37.9% were from Andalusia and 27.5% from Madrid.

The mean participant age was 40.87 (*SD* = 12.42), in a range of 18 to 76. The sample was made up of 67.2% (n = 681) women and 32.8% men, with a mean age of 39.88 (*SD* = 12.35) and 42.92 years (*SD* = 12.33), respectively.

Marital status was 60.1% (n = 609) married or with a stable partner, 30.9% (n = 313) single, 8.1% (n = 82) divorced or separated, and the remaining 1% (n = 10) widowed. 35.9% (n = 364) of the participants stated they had minors in their care.

With respect to education, 78.7% (n = 798) had a higher education, followed by 16% (n = 162) with secondary education, 5% (n = 51) with primary school and 0.3% had no formal education.

The whole sample, at the time data were collected, were ordered confined to their homes by the State of Emergency decreed by the government of Spain in response to the current COVID-19 pandemic. Of all those surveyed, 15.4% (n = 166) said they had a positive case of coronavirus infection close to them.

### Instruments

An ad hoc questionnaire was prepared for collecting sociodemographic characteristics. Items included sex, marital status, age, education and if there was anyone with coronavirus close to them.

The Brief Illness Perception Questionnaire (BIP-Q) [24], in the brief [25] Spanish version validated for COVID-19 [26] was used for this study. This instrument consists of five items answered on a 10-point Likert-type scale which finds a single dimension, perception of threat from the disease, for which reliability was ω = 0.68; GLB = 0.72.

Emotional state during the past week was evaluated with the Affective Balance Scale (ABS) [27]; adapted to a Spanish population [28]. This instrument consists of 18 items in which the subjects must say whether they have experienced the states indicated in the past week rated on a Likert-type scale with three answer choices on the frequency (1 = “little or never”, 2 = “Sometimes”, 3 = “A lot or usually”). The scale directly measures both positive and negative affect. The reliability indices found were ω = 0.82, GLB = 0.83 and ω = 0.79, GLB = 0.82, respectively.

Their emotional condition at the time of evaluation was analyzed with the Mood Evaluation Scale (EVEA) [29]. This instrument evaluates transitory moods, classifying them in four subscales (anxiety, sadness-depression, anger-hostility and joy). It has 16 items rated on a scale of 0 (none) to 10 (a lot) indicating how much the subject identifies with the different moods enumerated. Reliability was ω = 0.88, GLB = 0.89 for anxiety; ω = 0.88, GLB = 0.90 for sadness-depression; ω = 0.96, GLB = 0.97 for anger hostility; and ω = 0.85, GLB = 0.88 for joy.

### Procedure

This cross-sectional study was carried out in a sample found by snowball sampling, by publicizing it on social networks and by texting during the first week of confinement of the Spanish population from March 18 to 23, 2020. A CAWI (Computer Aided Web Interviewing) survey was used for data collection, including, in addition to the three validated questionnaires, a series of questions for collecting sociodemographic data (age, sex, marital status, education) and others on their current situation (minor children in their care, or positive coronavirus cases in their closest environment). Participation was voluntary, and before answering the questionnaire, participants were given information on the study and its purpose on the first page, where they also had to check a box indicating their informed consent before they could start taking the survey. They were also asked to answer sincerely, guaranteeing the anonymity of their answers. For control of random answers, a series of control questions were included throughout the questionnaire. The study was approved by the University of Almeria Bioethics Committee (Ref. Favorably reported on March 24, 2020).

### Data analysis

First, Pearson’s pairwise correlation coefficients were determined. Then the descriptive statistics for the variables of the study were presented.

Participants were grouped according to the Affective Balance Index (ABI), on which scores below 0 show a “negative affective balance” and above 0 a “positive affective balance”. A third group, which we called the “neutral group”, was made up of those whose score was equal to 0. A *t*-test for independent samples was performed to find out whether there were any differences between the groups (Negative and Positive Affective Balance) in perceived threat from the disease. Furthermore, the Bayesian alternative, which enables estimation of evidence in favor of the hypothesis using the Bayes Factor, was also calculated. JASP statistical software ver. 0.11.1 [30] was used for estimating the Bayesian *t*-test. The Cauchy prior width was 0.707 as predetermined by the software [31].

Starting out from the results of the correlation analyses, several mediation models were proposed. Specifically, two mediation analyses were done with predictors, mediators and multiple result variables. Model estimation was performed applying statistical corrections, including the item on the close presence of a COVID-19-positive case as a confusion variable. JASP ver. 0.11.1 [30] based on the lavaan software [32] was used for this. To prove whether there was an indirect effect, bootstrapping was applied, calculating the confidence intervals with the bias-corrected percentile method as suggested by Biesanz, Falk & Savalei [33].

To examine the reliability of the instruments used for data collection, McDonald’s Omega [34] coefficient was estimated, following the proposal and indications of Ventura-León and Caycho [35]. The Greatest Lower Bound (GLB) was also calculated.

## Results

### Preliminary analyses: Correlations and descriptive statistics

As shown in Table 1, perceived threat from the disease correlated positively with negative moods such as Sadness-Depression, Anxiety and Anger-Hostility, and on the contrary, was negatively correlated with the Joy subscale.

**Table 1:** Pearson correlations and descriptive statistics.

BIP-Q	EVEA / EBA	Pearson's r	Lower 95% CI	Upper 95% CI	Mean	Stand. Dev.
PERCEIVED THREAT M = 30.74, SD = 6.63	Sadness-Depression	0.47 ***	0.430	0.525	4.98	2.24
	Anxiety	0.60 ***	0.567	0.645	5.79	2.22
	Anger-Hostility	0.43 ***	0.388	0.488	4.81	2.62
	Joy	-0.18 ***	-0.245	-0.126	4.42	1.79
	Positive affect	-0.22 ***	-0.285	-0.168	16.31	3.71
	Negative affect	0.50 ***	0.452	0.545	16.16	3.70

*** p < .001

In relationships with affect, perceived threat from the disease correlated negatively with Positive affect and positively with Negative affect.

Moreover, classification of the ABI resulted in three groups: Negative Affective Balance, Positive Affective Balance and the group we called “neutral”, because they scored an ABI = 0. This group (n = 66) was discarded for the comparison of means, using only the differences between the Negative (n = 461) and Positive Affective Balance (n = 487).

**Fig 2:**
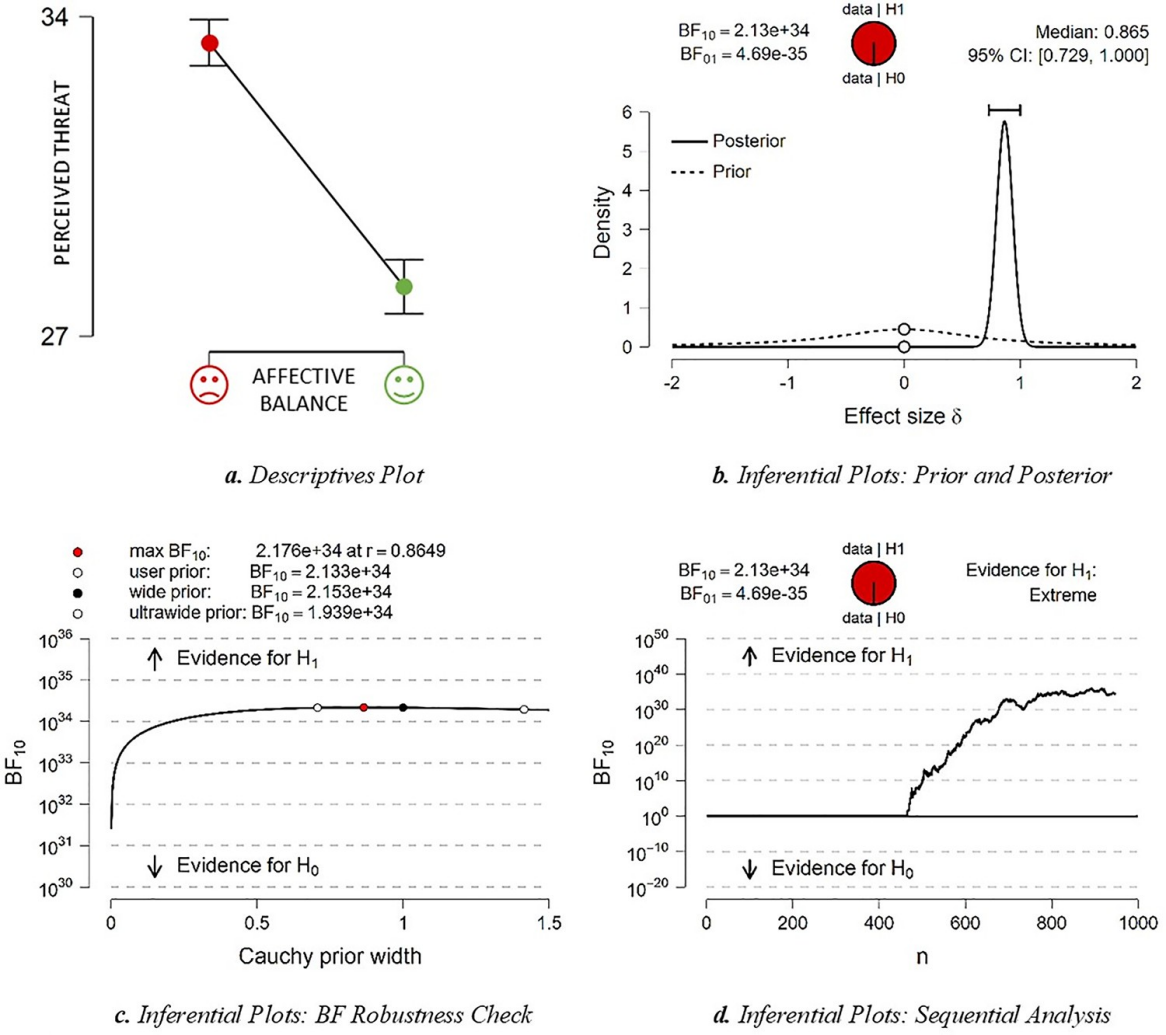
Descriptives and inferential plots. ☺ = Positive affect, ☹ = Negative affect.

Table 2 shows the statistically significant differences between the two groups, where the negative affective balance group had a higher mean score in perceived threat from the disease than the positive affective balance group (Fig 2A). The Cohen’s *d* of over 0.8 indicates a large effect size.

**Table 2:** Affective balance groups and independent samples t-test.

	Negative Affective Balance			Positive Affective Balance			*t*	Mean Dif.	SE Dif.	95% CI		Cohen's *d*
	*N*	*M*	*SD*	*N*	*M*	*SD*				Lower	Upper	
Perceived Threat	461	33.43	5.53	487	28.08	6.64	13.42***	5.34	0.39	4.564	6.127	0.87

The Bayes Factor (BF) was also computed to test the weight of available evidence in favor of the alternative hypothesis (H_1_) on the existence of differences between groups against the null hypothesis (H_0_) that there is no significant difference between groups. The data found on the differences between groups support the evidence in favor of the alternative hypothesis (BF_10_ = 2.133x10^34^), which revealed extreme evidence in favor of H_1_. The inference graphs corresponding to the Bayesian statistic are shown in Fig 2B, 2C and 2D.

### Mediation models

As a starting point, in line with the hypothetical circular model presented above (Fig 1) and to formulate the mediation models, we asked ourselves two questions: 1) How does perceived threat from COVID-19 affect mood? (Model 1), and 2) How does mood affect perceived threat from COVID-19? (Model 2). And in both cases, are positive and negative affect mediators in these relationships?

As observed in Table 3, the perception of threat had a direct positive effect on moods that could be qualified as “negative” (Sadness-Depression, Anxiety, Anger-Hostility).

**Table 3:** Direct, indirect and total effects (Model 1).

**Direct effects**					95% CI	
	Estimate	Std. Error	z-value	p	Lower	Upper
PT_COVID-19_ → S-D	0.171	0.026	6.688	< .001	0.121	0.221
PT_COVID-19_ → ANX	0.360	0.025	14.652	< .001	0.312	0.408
PT_COVID-19_ → A-H	0.256	0.031	8.349	< .001	0.196	0.316
PT_COVID-19_ → JOY	0.027	0.030	0.894	0.371	-0.032	0.086
**Indirect effects**					95% CI	
	Estimate	Std. Error	z-value	p	Lower	Upper
PT_COVID-19_ → ☺ → S-D	0.032	0.007	4.657	< .001	0.018	0.045
PT_COVID-19_ → ☹ → S-D	0.276	0.020	13.819	< .001	0.237	0.316
PT_COVID-19_ → ☺ → ANX	0.010	0.005	1.935	0.053	-1.323e -4	0.021
PT_COVID-19_ → ☹ → ANX	0.236	0.018	13.062	< .001	0.200	0.271
PT_COVID-19_ → ☺ → A-H	-2.099e -4	0.006	-0.033	0.974	-0.013	0.012
PT_COVID-19_ → ☹ → A-H	0.183	0.019	9.808	< .001	0.147	0.220
PT_COVID-19_ → ☺ → JOY	-0.106	0.016	-6.773	< .001	-0.137	-0.075
PT_COVID-19_ → ☹ → JOY	-0.105	0.017	-6.357	< .001	-0.138	-0.073
**Total effects**					95% CI	
	Estimate	Std. Error	z-value	p	Lower	Upper
PT_COVID-19_ → S-D	0.479	0.028	17.310	< .001	0.425	0.533
PT_COVID-19_ → ANX	0.606	0.025	24.202	< .001	0.557	0.655
PT_COVID-19_ → A-H	0.439	0.028	15.514	< .001	0.384	0.495
PT_COVID-19_ → JOY	-0.184	0.031	-5.954	< .001	-0.245	-0.124

PT_COVID-19_ = Perceived Threat COVID-19, S-D = Sadness-Depression, ANX = Anxiety, A-H = Anger-Hostility, JOY = Joy. ☺ = Positive affect, ☹ = Negative affect. Delta method standard errors, bias-corrected percentile bootstrap confidence intervals.

Indirect effects observed were that positive affect mediated in the relationship of perceived threat and Sadness-Depression and Joy. Negative affect exerted a mediating function in the relationship between perceived threat and the four moods, positive for Sadness-Depression, Anxiety, Anger-Hostility, and negative for Joy.

The proportion of variance explained for each of the predictor variables in mediation Model 1 is the following: 51% (*R*^*2*^ = 0.510) for Sadness-Depression, 54.7% (*R*^*2*^ = 0.547) for Anxiety, 29.3% (*R*^*2*^ = 0.293) for Anger-Hostility, and 31.9% (*R*^*2*^ = 0.319) for Joy. For the mediators we found 5.2% (*R*^*2*^ = 0.052) for Positive affect and 25% (*R*^*2*^ = 0.250) for Negative affect.

In Table 4, Anxiety and Anger-Hostility show a positive direct effect on perceived threat from COVID-19. As indirect effects observed, experiencing negative effect exerted a mediating role in the relationship between perceived threat and Sadness-Depression, Anxiety and Joy, negative in the last mood. There was no mediation by positive affect in any relationship.

**Table 4:** Direct, indirect and total effects (Model 2).

**Direct effects**					95% CI	
	Estimate	Std. Error	z-value	p	Lower	Upper
S-D → PT_COVID-19_	0.023	0.038	0.605	0.545	-0.052	0.098
ANX → PT_COVID-19_	0.431	0.039	11.030	< .001	0.354	0.508
A-H → PT_COVID-19_	0.088	0.032	2.761	0.006	0.025	0.150
JOY → PT_COVID-19_	0.036	0.030	1.218	0.223	-0.022	0.094
**Indirect effects**					95% CI	
	Estimate	Std. Error	z-value	p	Lower	Upper
S-D → ☺ → PT_COVID-19_	0.010	0.006	1.593	0.111	-0.002	0.022
S-D → ☹ → PT_COVID-19_	0.056	0.015	3.857	< .001	0.028	0.085
ANX → ☺ → PT_COVID-19_	0.003	0.003	1.217	0.223	-0.002	0.009
ANX → ☹ → PT_COVID-19_	0.050	0.013	3.787	< .001	0.024	0.076
A-H → ☺ → PT_COVID-19_	-0.004	0.003	-1.362	0.173	-0.010	0.002
A-H → ☹ → PT_COVID-19_	0.009	0.005	1.886	0.059	-3.348e -4	0.017
JOY → ☺ → PT_COVID-19_	-0.023	0.014	-1.660	0.097	-0.050	0.004
JOY → ☹ → PT_COVID-19_	-0.018	0.006	-3.280	0.001	-0.030	-0.007
**Total effects**					95% CI	
	Estimate	Std. Error	z-value	p	Lower	Upper
S-D → PT_COVID-19_	0.090	0.036	2.505	0.012	0.019	0.160
ANX → PT_COVID-19_	0.484	0.037	12.945	< .001	0.411	0.558
A-H → PT_COVID-19_	0.092	0.032	2.895	0.004	0.030	0.155
JOY → PT_COVID-19_	-0.005	0.026	-0.205	0.837	-0.057	0.046

PT_COVID-19_ = Perceived Threat COVID-19, S-D = Sadness-Depression, ANX = Anxiety, A-H = Anger-Hostility, JOY = Joy. ☺ = Positive affect, ☹ = Negative affect. Delta method standard errors, bias-corrected percentile bootstrap confidence intervals.

Finally, in Model 2, the proportion of explained variance of the predictor variable (perceived threat) is 39.4% (*R*^*2*^ = 0.394). For each of the mediators, we found 32.6% (*R*^*2*^ = 0.326) for Positive affect and 55.4% (*R*^*2*^ = 0.554) for Negative.

## Discussion

This study analyzed the cognitive and emotional (affective and mood) states of individuals in preventive quarantine. The results confirmed our starting hypothesis. First, they found that perceived threat from COVID-19 was related positively to negative affect and emotional states, that is sadness-depression, anxiety and anger-hostility, while the relationship shown with positive affect and feeling of joy was negative. According to the literature, confinement can cause severe consequences for psychological wellbeing [13], as negative feelings usually appear [8]. It is also an uncertain situation, when it is unclear where events will lead to, which causes high stress, and an increased perception of threat [16]. Strong perception of threat for one’s health can, in turn, cause an altered affective state and feelings of irritation, anxiety, despondency or sadness [9,18]^.^

Similarly, differences were found in perceived threat from the pandemic according to the affective balance during the previous week of confinement. The analyses found two opposite groups, one framed by positive affect and the other by negative. The latter showed a higher mean score in perceived susceptibility to disease, which coincides with previous literature [5].

Finally, a circular relationship, as also observed by other authors [17], was found, in which perceived threat influenced the presence of negative mood, and negative mood, in turn, was linked to irritation and nervousness in the current situation promoted by the individual’s feeling of threat. Thus, perceived vulnerability to contagion increased the individual’s perception of threat [7,16], which promoted negative mood [8,13], while emotions of hostility and distress, partly generated by the perception of threat from COVID-19 itself and sudden confinement [14], affected the cognitive state, increasing apprehension [18]. In both cases, the negative affective state mediated in this relationship.

This study had some limitations which should be mentioned. First, variables such as age or sex, which have been shown to be determining in the evolution of the virus, were not taken into account. Both sociodemographic variables could be closely related to perceived threat from COVID-19. Since most of the sample were women and the mean age has not been associated with high mortality, the model’s generalization to the overall population would have to be tested. Neither were COVID-19 symptoms or diagnosis in the individual or in close friends and family, or perceived health condition considered, which could have affected the perception of threat from the disease. It should also be mentioned that in spite of the large number of studies on the variables dealt with here in highly stressful situations, there are no previous studies analyzing them together with mediation models, which in turn, decreases the possibility of comparing our results. In addition, it should be mentioned that the data were taken at the beginning of the quarantine, specifically, during the second week of confinement. Thus, the results of this study should be taken with caution, since the emotions shown and perception of risk during this initial period may have changed over time as the confinement decree was extended and the virus spread.

### Practical applications

The current COVID-19 health emergency has completely changed the daily life of the Spanish population. Both the confinement scenario and the spread of the virus, as well as associated consequences could cause alteration of people’s cognitive and emotional state through perceived threat from the virus and development of negative affective balance and feelings. Therefore, knowledge of the variables associated with the development of these alterations is fundamental to prevention and coping with confinement in similar populations and in the context following the pandemic, where recovery of psychological wellbeing will become a primary goal. The results of this study show how perceived threat is a risk variable for development of negative mood and vice versa, operating as a mediator in this circular relationship of negative affective balance, which increases both effects.

As the most effective healthcare measure for reducing the incidence of the coronavirus pandemic is quarantine, and globalization and travel of the population facilitate the probability of similar situations occurring again, knowledge of the emotional and cognitive effects on the population could enable measures that facilitate their more effective coping to be put to use. The results of this study will be put into practice with the implementation of a psychoeducational program for working on the emotional state (EMOCOVID). Thus, in view of the affective and emotional alterations found during the quarantine and their relationship to perceived threat, it is intended to design a program to facilitate emotional management with daily activities related to knowledge, connection and management of emotions that could appear during quarantine.

## Supporting information

S1 Checklist STROBE statement—checklist of items that should be included in reports of *cross-sectional studies*. (DOCX)

S1 Data (SAV)

## References

[1] Hellewell J, Abbot S, Gimma A, Bosse NI, Jarvis CI, Russell TW, et al Feasibility of controlling COVID-19 outbreaks by isolation of cases and contact. *Lancet Glob Health*. 2020; 8: e488–96. 10.1016/S2214-109X(20)30074-7 32119825 PMC7097845

[2] Sohrabi C, Alsafi Z, Neill NO, Khan M, Kerwan A, Al-Jabir A, et al World Health Organization declares global emergency: A review of the 2019 novel coronavirus (COVID-19). *Int J Surg*. 2020; 76: 71–6. 10.1016/j.ijsu.2020.02.034 32112977 PMC7105032

[3] Ministry of Health. Infection prevention and control in the management of patients with COVID-19 [Prevención y control de la infección en el manejo de pacientes con COVID-19]. [Internet]. Madrid, España: Ministerio de Sanidad, 2020 Available from: https://www.mscbs.gob.es/profesionales/saludPublica/ccayes/alertasActual/nCov-China/documentos/Documento_Control_Infeccion.pdf

[4] Bauer EA, Braitman AL, Judah MR, Cigularov KP Worry as a mediator between psychosocial stressors and emotional sequelae: Moderation by contrast avoidance. *J Affect Disord*. 2020; 266: 456–64. 10.1016/j.jad.2020.01.092 32056913

[5] Jin Y, Austin L, Vijaykumar S, Jun H, Nowak G. Communicating about infectious disease threats: Insights from public health information officers. *Public Relat*. *Rev*. 2019; 45(1): 167–77.

[6] Zhang TK. Factors influencing seasonal influenza vaccination behaviour among elderly people: a systematic review. *Public Health*. 2018; 156: 67–78. 10.1016/j.puhe.2017.12.007 29408191 PMC7111770

[7] Chang BP. Can hospitalization be hazardous to your health? A nosocomial based stress model for hospitalization. *Gen*. *Hosp*. *Psychiatry*. 2019; 60, 83–9. 10.1016/j.genhosppsych.2019.07.014 31376645 PMC6791742

[8] Moreira de Medeiros P, Moreno M, Nogueira M, Marcondes J, Leite M. The psychiatric impact of the novel coronavirus outbreak. *Psychiatry Res*. 2020; 286: 112902 10.1016/j.psychres.2020.112902 32146248 PMC7133679

[9] Park SC, Park YC. Mental Health Care Measures in Response to the 2019 Novel Coronavirus Outbreak in Korea. *Psychiatry Investig*. 2020; 17(2): 85–6. 10.30773/pi.2020.0058 32093458 PMC7047003

[10] Royal Decree 463/2020, of March 14, declaring the state of alarm for the management of the health crisis situation caused by COVID-19, of March 18, 2020 [Real Decreto 463/2020, de 14 de marzo, por el que se declara el estado de alarma para la gestión de la situación de crisis sanitaria ocasionada por el COVID-19, de 18 de marzo de 2020], 25944 a 25945. Available from: https://www.boe.es/buscar/doc.php?id=BOE-A-2020-3828

[11] Lombardi A, Bozzi G, Mangioni D, Muscatello A, Peri AM, Taramasso L, et al Duration of quarantine in hospitalized patients with severe acute respiratory syndrome coronavirus 2 (SARS-CoV-2) infection: a question needing an answer. *J Hosp Infect*. 2020; In Press. 10.1016/j.jhin.2020.03.003 PMC7134399 32151674

[12] Khot WY, Nadkar MY. The 2019 Novel Coronavirus Outbreak—A Global Threat. *J Assoc Physicians India*. 2020; 68(3): 67–71. 32138488

[13] Wang C, Pan R, Wan X, Tan Y, Xu L, Ho CS, et al Immediate Psychological Responses and Associated Factors during the Initial Stage of the 2019 Coronavirus Disease (COVID-19) Epidemic among the General Population in China. *Int*. *J*. *Environ*. *Res*. *Public Health*. 2020; 17(5): 1729. 10.3390/ijerph17051729 PMC7084952 32155789

[14] Brooks SK, Webster RK, Smith LE, Woodland L, Wessely S, Greenberg N, et al The psychological impact of quarantine and how to reduce it: rapid review of the evidence. *Lancet*. 2020; 395(10227): 912–20. 10.1016/S0140-6736(20)30460-8 32112714 PMC7158942

[15] Vicente de Vera MI, Gabari MI. Resilience as a protective factor of chronic stress in teacher. *Eur J Investig Health Psychol Educ*, 2019; 9(3): 159–75.

[16] Cisler JM, Koster EH. Mechanisms of attentional biases towards threat in anxiety disorders: An integrative review. *Clin*. *Psychol*. *Rev*. 2010; 30(2): 203–16. 10.1016/j.cpr.2009.11.003 20005616 PMC2814889

[17] Macatee RJ, Albanese BJ, Schmidt NB, Cougle JR. Attention bias towards negative emotional information and its relationship with daily worry in the context of acute stress: an eye-tracking study. *Behav Res Ther*. 2017; 90: 96–110. 10.1016/j.brat.2016.12.013 28013055 PMC5346289

[18] Sapolsky RM. Stress, Health and Social Behavior In: Choe JC, editor. Animal Behavior. Elsevier; 2019 p. 163–70.

[19] Molero MM, Pérez-Fuentes MC, Gázquez JJ, Simón MM, Martos Á. Burnout Risk and Protection Factors in Certified Nursing Aides. *Int*. *J*. *Environ*. *Res*. *Public Health*. 2018; 15(6): 1116. 10.3390/ijerph15061116 PMC6025175 29848982

[20] Molero MM, Pérez-Fuentes MC, Gázquez JJ, Barragán AB. Burnout in Health Professionals According to Their Self-Esteem, Social Support and Empathy Profile. *Front Psychol*. 2018; 9: 424 10.3389/fpsyg.2018.00424 29731725 PMC5920032

[21] Pérez-Fuentes MC, Molero MM, Gázquez JJ, Simón MM. Analysis of Burnout Predictors in Nursing: Risk and Protective Psychological Factors. *Eur J Psychol Appl L*. 2019; 11(1): 33–40.

[22] Vizoso-Gómez C, Arias-Gundín O. Resilience, optimism and academic burnout in university students. *Eur*. *j*. *educ*. *psycho*. 2018; 11(1): 47–59.

[23] Castellano E, Muñoz-Navarro R, Toledo MS, Spontón C, Medrano LA. Cognitive processes of emotional regulation, burnout and work engagement. *Psicothema*. 2019; 31(1): 73–80. 10.7334/psicothema2018.228 30664414

[24] Broadbent E, Petrie KJ, Weinman J. The brief illness perception questionnaire. *J Psychosom Res*. 2006; 60(6): 631–7. 10.1016/j.jpsychores.2005.10.020 16731240

[25] Valero-Moreno S, Lacomba-Trejo L, Casaña-Granell S Prado-Gascó VJ, Montoya-Castilla I, Pérez-Marín M. Psychometric properties of the Questionnaire on Threat Perception of Chronic Illnesses in pediatric patients. *Rev Lat Am Enfermagem*. 2020; 28: e3242 10.1590/1518-8345.3144.3242 32022154 PMC7000189

[26] Pérez-Fuentes MC, Molero MM, Oropesa NF, Herrera-Peco I, Gázquez JJ. Questionnaire on perception of threat from COVID-19. *J Clin Med*. 2020. 10.3390/jcm9041196 PMC7230235 32331246

[27] Warr P, Barter J, Brownbridge G. On the independence of positive and negative affect. *J*. *Pers*. *Soc*. *Psychol*. 1983; 44: 644–51.

[28] Godoy-Izquierdo D, Martínez A, Godoy JF. The «Affective Balance Scale». Psychometric properties of an instrument to measure positive and negative affect in the Spanish population [La «Escala de Balance Afectivo». Propiedades psicométricas de un instrumento para la medida del afecto positivo y negativo en población española]. *Clínica y Salud*. 2008; 19(2): 175–89.

[29] Sanz J. An instrument to evaluate the efficacy of mood induction procedures: the “Mood Assessment Scale” (EVEA) [Un instrumento para evaluar la eficacia de los procedimientos de inducción de estados de ánimo: la “Escala de Valoración del estado de Ánimo” (EVEA)]. *‎* *Anál*. *Modif*. *Conduct*. 2001; 27(111): 71–110.

[30] JASP. Team JASP (Version 0.11.1) [Computer software]; 2019.

[31] Morey RD, Rouder JN. BayesFactor 0.9.12–2. Comprehensive R Archive Network; 2015.

[32] Rosseel Y. Lavaan: An R package for structural equation modeling and more. Version 0.5–12 (BETA). *J Stat Softw*. 2012; 48(2): 1–36.

[33] Biesanz JC, Falk CF, Savalei V. Assessing Mediational Models: Testing and Interval Estimation for Indirect Effects. *Multivar Behav Res*. 2010; 45(4): 661–701. 10.1080/00273171.2010.498292 26735714

[34] McDonald RP. Test theory: A unified approach. Mahwah, NJ: Lawrence Erlbaum Associates; 1999.

[35] Ventura-León JL, Caycho T. The Omega coefficient: an alternative method for estimating reliability [El coeficiente Omega: un método alternativo para la estimación de la confiabilidad]. *Rev Latinoam Cienc Soc Niñez Juv*. 2017; 15: 625–7.

